# Application of immunoPET imaging to enhance head and neck squamous cell carcinoma clinical management

**DOI:** 10.3389/fonc.2025.1644692

**Published:** 2025-10-23

**Authors:** Waleed M. Almutairi, Qi-Huang Zheng, Mark Langer, Oluwaseyi M. Oderinde

**Affiliations:** ^1^ Advanced Molecular Imaging in Radiotherapy (AdMIRe) Research Laboratory, Purdue University, West Lafayette, IN, United States; ^2^ Department of Medical Imaging, King Abdullah bin Abdulaziz University Hospital, Princess Nourah bint Abdulrahman University, Riyadh, Saudi Arabia; ^3^ Department of Radiology, Indiana University School of Medicine, Indianapolis, IN, United States; ^4^ Department of Radiation Oncology, Indiana University School of Medicine, Indianapolis, IN, United States

**Keywords:** immunoPET, tumor targeting, head and neck squamous cell carcinoma (HNSCC), radiolabeled antibodies, personalized medicine

## Abstract

**Background:**

Head and neck squamous cell carcinoma (HNSCC) remains a significant clinical challenge due to high recurrence, therapy resistance, and limited biomarkers. The tumor microenvironment (TME) plays a critical role in determining treatment outcomes. Immuno-positron emission tomography (immunoPET), which combines the specificity of monoclonal antibodies (mAbs) with the sensitivity of PET, offers non-invasive visualization of immune activity and guidance for treatment. This review summarizes the applications of immunoPET in HNSCC.

**Methods:**

Followed PRISMA 2020 guidelines, 1686 records were identified through searches of PubMed, Embase, Scopus, and Web of Science (January 1, 1999, to May 11, 2025). Only 11 studies on immunoPET in HNSCC met the inclusion criteria and were evaluated for imaging targets, radiotracers, injection approaches, and preclinical or clinical outcomes. Methodological quality was assessed using the Quality Assessment of Diagnostic Accuracy Studies (QUADAS-2) and the Systematic Review Center for Laboratory Animal Experimentation (SYRCLE) tools.

**Results:**

Six preclinical and five clinical studies investigated five immune-related targets: programmed death-ligand 1 (PD-L1), epidermal growth factor receptor (EGFR), tenascin-C, the extra domain B (ED-B) of fibronectin, and cluster of differentiation 44 variant 6 (CD44v6). PD-L1 imaging demonstrated safety and feasibility but lacked predictive accuracy. EGFR imaging showed high preclinical receptor-specific specificity, whereas clinical performance revealed cetuximab tumor accessibility, which was undetectable by FDG PET, with significant variability between patients. Tenascin-C imaging was localized to tumors but missed some lymph node metastases. ED-B imaging visualized tumor angiogenesis and reliably predicted therapeutic biodistribution, while CD44v6 emerged as the most consistently evaluated and promising for clinical translation.

**Conclusion:**

ImmunoPET holds promise for patient stratification and early response monitoring in HNSCC. Evidence remains limited, primarily due to small cohorts, heterogeneous protocols, narrow target diversity, and reliance on long-lived tracers. Future research should broaden the immune target, optimize imaging protocols, and develop short-lived tracers (e.g., ^18^F, ^68^Ga) for broader clinical integration.

## 1 Introduction

Head and neck squamous cell carcinoma (HNSCC) is the sixth most prevalent malignant tumor, with a rising global incidence (1), and over 480, 000 deaths annually (2). A delay of approximately four weeks in initiating treatment can result in significant tumor progression (3). Despite therapeutic and research advances, the 5-year survival rates for HNSCC remain low, primarily due to metastasis, recurrence, therapy resistance, and late diagnosis (4). A significant current challenge in HNSCC management is the lack of prognostic biomarkers beyond human papillomavirus (HPV) status in clinical practice (1). Moreover, conventional therapies often overlook the immune activities in the tumor microenvironment (TME) (5), which play a pivotal role in tumor progression and therapeutic resistance (6). The dynamics of immune cell subpopulations are crucial for determining treatment response or resistance (7), underscoring the importance of understanding immune cell activity within the body (8).

Several studies have highlighted the importance of the immune activities in TME influencing treatment response and prognosis in HNSCC. Various immune-related biomarkers have demonstrated prognostic significance. Specifically, increased infiltration of CD8+ T cells and natural killer (NK) cells has been associated with improved outcomes. In contrast, elevated levels of immunosuppressive cells (including regulatory T cells, myeloid-derived suppressor cells (MDSCs), neutrophils, and M2 macrophages) are associated with poor prognosis (9).

Treatment efficacy is commonly evaluated using tumor volume measurements, post-treatment tissue biopsies, or blood-based assays, although each method has its drawbacks (10). Tumor enlargement may not indicate the actual spatial aggressiveness of the disease, as an influx of helpful immune cells to the TME often contributes to increased volume, known as pseudoprogression. Post-therapy biopsies are invasive, limited by tumor accessibility, often miss tumor heterogeneity, and may affect the surrounding TME and hinder the patient’s consent. Blood-based assays remain unstandardized, often depend on prior knowledge of specific tumor antigens, and may not reflect immune activity within the tumor (10). Additionally, biopsies cannot capture the heterogeneous distribution of immune cells across all tumor sites and accurately track the response to treatment (11). Thus, there is a pressing clinical need for non-invasive diagnostic and predictive approaches to identify immune activities in the TME (10). Immune-based molecular imaging offers the potential to overcome several limitations associated with biopsy-based techniques, including the need for invasive sampling, limited tissue availability, and spatial heterogeneity (12, 13).

^18^F-fluorodeoxyglucose (FDG) is the most widely used positron emission tomography (PET) tracer, playing a critical role in clinical oncology (14, 15) across various cancers (16–19). It captures regions with increased metabolic activity in the body due to a similarity with glucose. However, despite its widespread application, ^18^F-FDG-PET has several limitations. Physiological uptake in normal tissues complicates image interpretation, and inflammatory conditions can lead to false positives, thereby reducing diagnostic specificity (20, 21). Due to their similar uptake patterns, FDG uptake cannot distinguish between proliferating tumor cells and activated immune cells (7). Moreover, distinguishing FDG uptake from tumor growth versus immune activation remains challenging (22). Post-treatment FDG PET/CT scans with 12 weeks have approximately a 50% false-positive rate, often leading to unnecessary interventions (23). In response, alternative PET radiotracers have been extensively investigated to overcome the challenges of predicting and monitoring therapeutics (24). To address the limitations of FDG PET, immune-specific PET imaging has been developed to target either general immune-related markers or markers uniquely expressed during immune activation. These include radiotracers for general immune-related markers, such as CD8+ T cells and Programmed Death-Ligand 1 (PD-L1), as well as probes for enzymes involved in T cell activation, including ^18^F-arabinosyl guanine (^18^F-FAraG). Specifically highlighting activated immune cells within the TME, it can help differentiate them from resting immune cells and provide complementary information to FDG PET/CT for assessing immune responses (25).

However, there is currently no clear consensus on the distinction between the terms “immunoPET” and “immunePET” which are often used interchangeably in the literature despite referring to different antibody-based versus broader immune-targeted imaging. This lack of standardized nomenclature can lead to confusion in the interpretation of study designs and imaging objectives.

**Figure 1:**
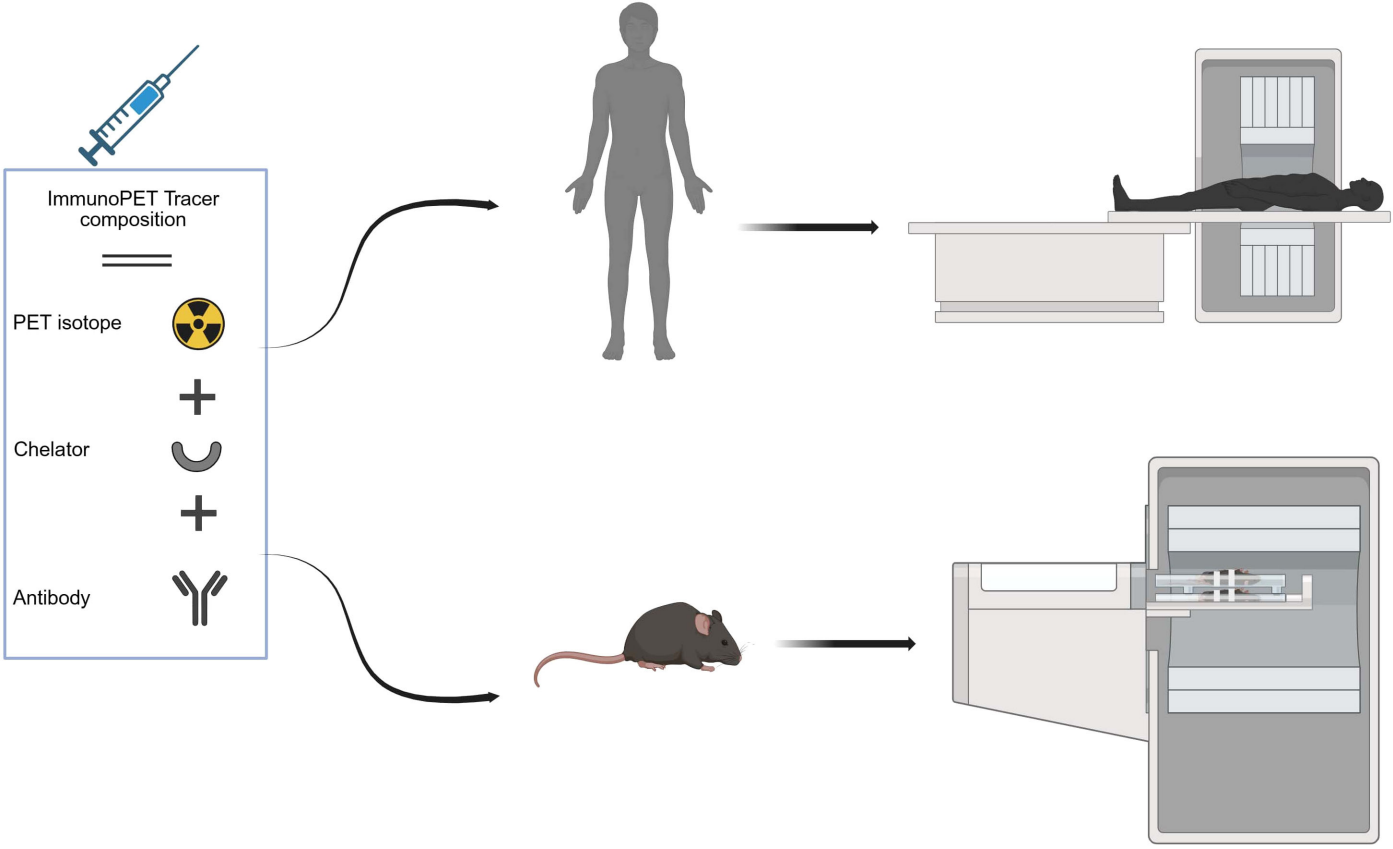
ImmunoPET tracer composition (isotope, chelator, antibody) and workflow for clinical and preclinical imaging. Created in BioRender. almutairi, w. (2025) https://BioRender.com/ntn1m4k.

Additionally, immunoPET is one of the most promising advancements in molecular imaging (26), which integrates monoclonal antibodies (mAbs) with PET radiotracers (26–28) to achieve non-invasive, whole-body, real-time 3D imaging of tumor biomarkers (26) and enables the quantitative assessment of functional parameters within the TME at the molecular level (29). A typical immunoPET tracer consists of a PET isotope, a chelator, and a targeting antibody, each playing a critical role in tumor localization and signal generation. By combining the mAb’s specificity with the PET’s sensitivity, immunoPET enables the precise detection of disease-associated biomarkers (27). Due to their target specificity and immune system activation, mAbs have gained prominence in oncology, infectious diseases, and inflammatory disorders (15). ImmunoPET is also increasingly being explored for these applications (30), with comparable workflows adapted for both preclinical and clinical studies (**Figure 1**). Beyond diagnosis, it monitors treatment response, particularly in cases where lesions are inaccessible, thereby reducing the need for invasive follow-ups like biopsies. It provides real-time insights into treatment efficacy and supports theragnostic applications, tumor classification, and personalized antibody-based therapies by visualizing immune responses to antibody-based therapies (26). First introduced in the literature in 1999, immunoPET has since emerged as a promising prognostic tool for assessing tumor heterogeneity and predicting therapy response, offering higher imaging resolution than conventional nuclear medicine techniques (15).

Given that only 20%-40% of cancer patients achieve a complete pathological response (31), early and accurate prediction of therapeutic effectiveness remains critical. This review aims to explore medical imaging strategies for enhancing risk stratification and treatment response, propose future research directions, and identify areas of deficiency and potential failure sites in HNSCC. This review examines the role of immunoPET in HNSCC, focusing on key radiotracers, clinical applications, and prospects. It highlights how immunoPET could improve treatment monitoring and tumor characterization compared with conventional approaches.

## 2 Methods

### 2.1 Search strategy

This review article was conducted according to the Preferred Reporting Items for Systematic Reviews and Meta-Analyses (PRISMA) 2020 guidelines (32) to ensure clarity and completeness in reporting. A comprehensive literature search was conducted in PubMed, Embase, Scopus, and the Web of Science Core Collection for studies published between January 1, 1999, and May 11, 2025. The search focused on studies related to immunoPET imaging in HNSCC. To ensure rigor, only free-text terms were used in the search strategy. These included variations and combinations such as “immunoPET” “immunePET” “HNSCC” “head and neck squamous cell carcinoma” “head and neck cancer” and “head and neck malignancy”. Records were screened based on titles, abstracts, and keywords. Subsequently, the authors manually reviewed the full texts to confirm eligibility and relevance.

### 2.2 Inclusion and exclusion criteria

Only studies published in English or translated into English were included. Studies involving cancers other than HNSCC or mixed cohorts beyond HNSCC or non-original articles (e.g., reviews) were intended to be excluded. However, no prior review articles specifically addressing immunoPET in HNSCC were identified during the search.

### 2.3 Quality assessment

The methodological quality of all included studies was evaluated using established tools appropriate to each study type. For clinical diagnostic accuracy studies (n = 5), assessment was conducted using the Quality Assessment of Diagnostic Accuracy Studies-2 (QUADAS-2) tool, a widely used and recommended tool for systematic reviews of diagnostic accuracy (33). QUADAS-2 evaluates four domains: patient selection, index test, reference standard, and flow and timing, to assess both risk of bias and concerns regarding applicability to the review question. Each domain is supported by signaling questions, and judgments are assigned as “low” “high” or “unclear” risk according to standardized criteria. For preclinical animal studies (n = 6), methodological rigor and risk of bias were assessed using the Systematic Review Center for Laboratory Animal Experimentation (SYRCLE) tool (34). This tool is an adaptation of the Cochrane risk of bias tool, designed to address unique aspects of animal experimentation. SYRCLE’s tool encompasses ten domains, including six types of bias: selection, performance, detection, attrition, reporting, and others. Each domain contains specific criteria that help to assess potential biases in study design, conduct, and reporting. The risk of bias for each item was graded as “low,” “high,” or “unclear”.

## 3 Results

**Figure 2:**
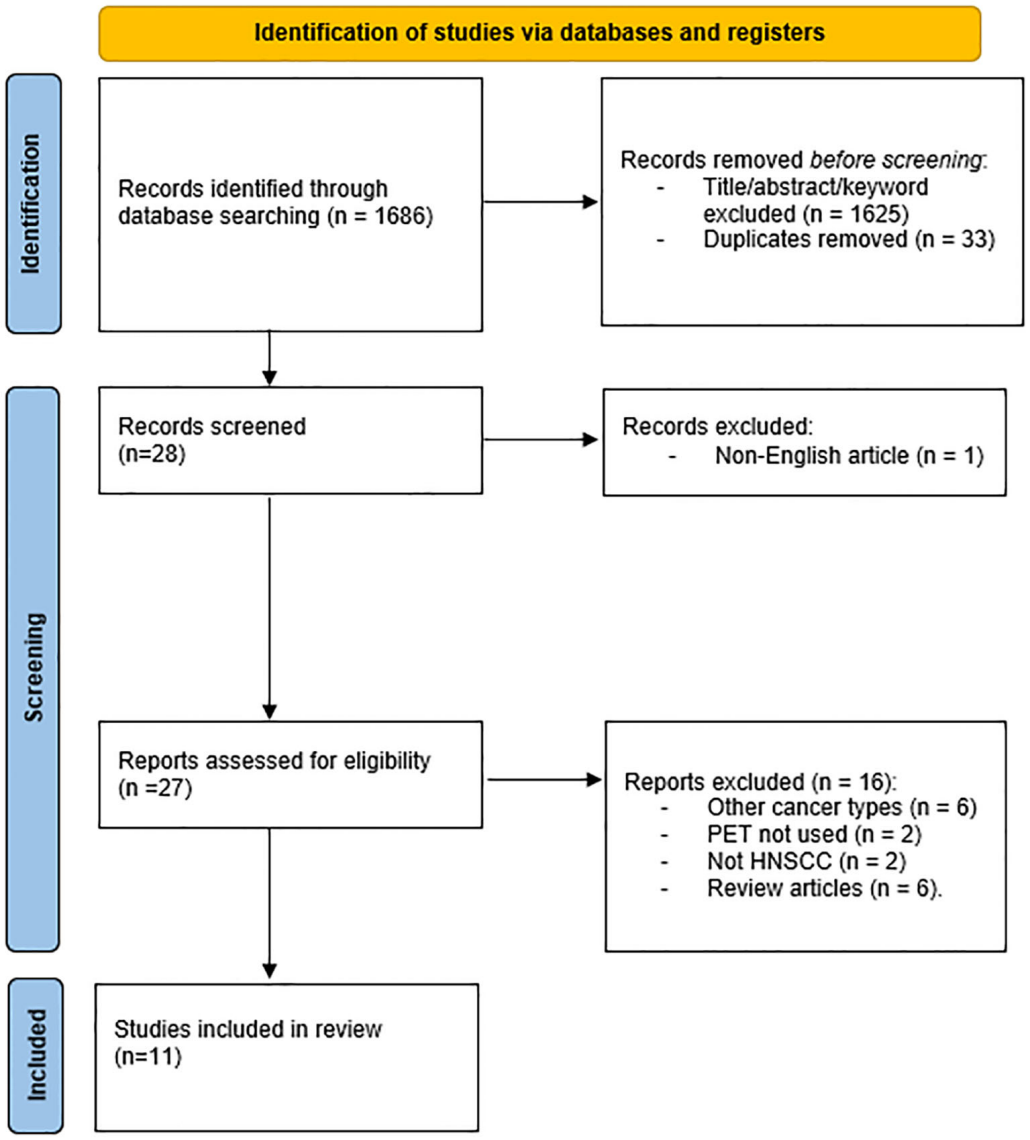
PRISMA flow diagram showing the study selection process. Of 1686 identified records, five studies met the final inclusion criteria after duplicate removal, screening, and eligibility assessment.

A total of 1, 686 records were initially identified. After removing duplicates and excluding articles that did not meet the date criteria, 28 articles remained for further screening. Following the eligibility assessment and application of inclusion criteria, 11 studies (six pre-clinical and five clinical) met the final inclusion criteria and were included in this review (**Figure 2**). These studies were evaluated for key characteristics, including radiotracers used, injected dose, treatment regimen, condition, molecular target, country of origin, study highlights, and the number of subjects (**Table 1**).

**Table 1:** Summary of included studies on immunoPET imaging in HNSCC.

Reference	Study type	Radiotracer	Chelator	Injected activity (MBq)	Tumor type	Target	Key findings	Country
Verhoeff et al., 2022 (35)	Clinical (n=33)	^89^Zr- durvalumab	DFO	37 MBq	Recurrent and metastatic HNSCC	PD-L1	Safe and feasible; no correlation with PD-L1 expression or response	Netherlands
Song et al., 2017 (38)	Preclinical (n=4)	^64^Cu-cetuximab	PCTA	3.7 MBq	HNSCC	EGFR	EGFR-specific accumulation and correlation with EGFR expression; supports feasibility in cetuximab-resistant HNSCC	Korea
Even et al., 2016 (39)	Clinical (n=17)	^89^Zr-cetuximab	DFO	54.5 ± 9.6 MBq	Stage III -IV, LA HNSCC	EGFR	Inter-patient uptake variability, partial correlation with EGFR; may aid tumor selection and outcome prediction	Netherlands
Heuveling et al., 2013 (40)	Clinical (n=4)	^124^I-F16SIP	None	71 ± 1.5 MBq	HNSCC	Tenascin-C	Safe, favorable pharmacokinetics, selective tumor uptake; potential for targeted imaging and therapy	Netherlands
Tijink et al., 2009 (42)	Preclinical (n=9)	^124^I-L19SIP	None	3.7 MBq	HNSCC	ED-B	^124^I-L19-SIP immunoPET enabled clear visualization of small angiogenic tumors (~50 mm³) and reliably predicted the biodistribution of ¹³¹I-L19-SIP for RIT	Netherlands
Verel et al., 2003 (44)	Preclinical (n=12)	^89^Zr-chimeric mAb U36	DFO	3.7 MBq	HNSCC	CD44v6	An ^89^Zr-labeling approach was developed, underscoring its potential for clinical immunoPET, combined with RIT for sensitive tumor detection and patient stratification	Netherlands
Verel et al., 2003 (45)	Preclinical (n=12)	^89^Zr-chimeric mAb U36	DFO	3.7 MBq	HNSCC	CD44v6	^89^Zr-U36 visualized tumors and strongly matched ^88^Y biodistribution, supporting ^89^Zr-PET as an *in-vivo* scout for ^90^Y RIT	Netherlands
Verel et al., 2004 (46)	Preclinical (n=8)	^124^ I-chimeric mAb U36	None	3.7 MBq	HNSCC	CD44v6	^124^I-U36 demonstrated effective tumor targeting; concordant ^124^I/¹³¹I biodistributions support ^124^I immunoPET as a scouting tracer for ¹³¹I RIT	Netherlands
Lumen et al., 2022 (43)	Group 1 (Targeted) Preclinical (n=4)	[^89^Zr]Zr-3–TCO–U36	DFO	4.4 ± 0.4 MBq	HNSCC	CD44v6	High tumor uptake and good contrast; used as reference for pretargeting	Netherlands
	Group 2 (Pretargeted-24h) Preclinical (n=4)	[^89^Zr]Zr-PEG_5_-Tz	DFO	4.1 ± 0.3 MBq	HNSCC	CD44v6	Lower tumor uptake, reduced off-target dose; enabled precise PET imaging	Netherlands
	Group 3 (Pretargeted-48h) Preclinical (n=4)	[^89^Zr]Zr-PEG _5_-Tz	DFO	3.9 ± 0.5 MBq	HNSCC	CD44v6	Similar contrast to 24h, with further reduced off-target radiation	Netherlands
Börjesson et al., 2006 (47)	Clinical (n=20)	^89^Zr-chimeric mAb U36	DFO	74.9 ± 0.6 MBq	HNSCC	CD44v6	^89^Zr-chimeric mAb U36 immunoPET showed diagnostic performance comparable to CT/MRI, and matched ^18^F-FDG PET in accuracy, highlighting the strong clinical potential of CD44v6-targeted imaging	Netherlands
Börjesson et al., 2009 (48)	Clinical (n=20)	^89^Zr-cmAb U36	DFO	74.9 ± 0.6 MBq	HNSCC	CD44v6	^89^Zr-cmAb U36 immunoPET provides reliable, quantitative biodistribution data for patient stratification and treatment planning, while having a high effective dose	Netherlands

HNSCC, head and neck squamous cell carcinoma; immunoPET, immuno-positron emission tomography; DFO, desferrioxamine; PD-L1, programmed death-ligand 1; ^89^Zr, zirconium-89; ^64^Cu, copper-64; PCTA, propylene cross-bridged tetraazamacrocyclic chelator; EGFR, epidermal growth factor receptor; LA, locally advanced; ^124^I, iodine-124; Tenascin-C, extracellular matrix glycoprotein tenascin-C; ED-B, extra domain B of fibronectin; RIT, radioimmunotherapy; mAb, monoclonal antibody; cmAb, chimeric monoclonal antibody; CD44v6, cluster of differentiation 44 variant 6; PEG_5_-Tz, polyethylene glycol-5 linked tetrazine; TCO, trans-cyclooctene; Tz, tetrazine; ^131^I, iodine-131; ^88^Y, yttrium-88; CT, computed tomography; MRI, magnetic resonance imaging; FDG, fluorodeoxyglucose; ^18^F, fluorine-18.

**Figure 3:**
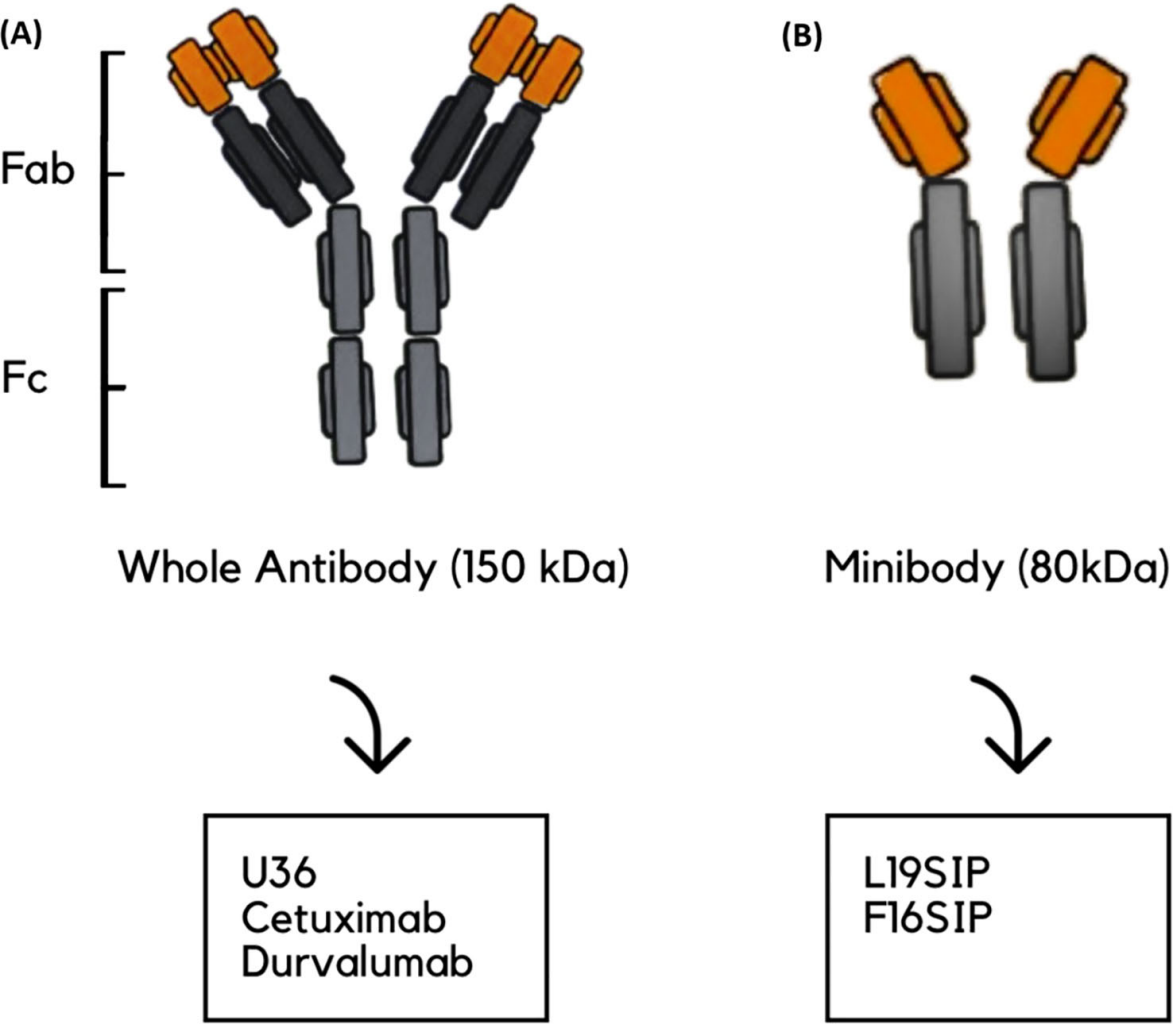
Schematic representation of antibody types used in the reviewed studies. **(A)** Whole antibody (~150 kDa), composed of Fab (fragment antigen-binding) and Fc (fragment crystallizable) regions; examples include U36, cetuximab, and durvalumab. **(B)** Minibody (~80 kDa); example: L19-SIP and F16SIP. **(A)** is shown on the left and **(B)** on the right.

Eleven studies explored the use of immunoPET imaging in the preclinical and clinical contexts of HNSCC, each targeting specific immune-related biomarkers: PD-L1, epidermal growth factor receptor (EGFR), tenascin-c, extra domain b (ED-B), and cluster of differentiation 44 variant 6 (CD44v6), all within the context of HNSCC. These studies used radiolabeled mAbs to visualize tumors, assess target expression, and characterize heterogeneity in uptake and therapeutic response. The antibodies used included full-size immunoglobulin G (IgG) antibodies, such as cetuximab, durvalumab, and U36, as well as smaller engineered fragments like L19 small immunoprotein (SIP) and F16 SIP (**Figure 3**), each offering distinct advantages in pharmacokinetics and tissue penetration.

As summarized in (**Table 2**), all clinical studies were rated as low risk for patient selection and across all applicability domains, supporting confidence in study populations and generalizability. In contrast, the index test domain was consistently assigned a high-risk rating due to the lack of a pre-specified threshold. The reference standard showed mixed results, with three studies classified as low risk and two as having unclear risk. For flow and timing, assessments were also varied, with two studies rated as low risk and three as high risk. In preclinical investigations, as detailed in (**Table 3**), several domains were consistently rated as unclear risk of bias due to insufficient reporting. These include sequence generation (D1), allocation concealment (D3), blinding (D5), random outcome assessment (D6), and blinding of outcome assessor (D7), primarily reflecting insufficient reporting. Baseline characteristics (D2), incomplete outcome data (D8), selective outcome reporting (D9), and other sources of bias (D10) were judged as low risk. Random housing (D4) was mixed, with two studies rated as low risk and three as unclear. Overall, all preclinical studies were deemed to have an unclear risk of bias, primarily due to a lack of methodological detail across several key domains.

**Table 2:** Assessment of risk of bias in clinical studies according to the QUADAS-2 tool.

First author, year (ref)	Patient selection	Index test	Reference standard	Flow and timing	Patient selection (applicability)	Index test (applicability)	Reference standard (applicability)
Börjesson et al., 2006 (47)	Low	High	Low	Low	Low	Low	Low
Börjesson et al., 2009 (48)	Low	High	Low	High	Low	Low	Low
Heuveling et al., 2013 (40)	Low	High	Unclear	Low	Low	Low	Low
Even et al., 2016 (39)	Low	High	Low	High	Low	Low	Low
Verhoeff et al., 2022 (35)	Low	High	Unclear	High	Low	Low	Low

**Table 3:** Assessment of risk of bias animal studies according to SYRCLE tool.

First author, year (ref)	D1	D2	D3	D4	D5	D6	D7	D8	D9	D10	Overall
Verel et al., 2003 (44)	Unclear	Low	Unclear	Unclear	Unclear	Unclear	Unclear	Low	Low	Low	Unclear
Verel et al., 2003 (45)	Unclear	Low	Unclear	Unclear	Unclear	Unclear	Unclear	Low	Low	Low	Unclear
Verel et al., 2004 (46)	Unclear	Low	Unclear	Unclear	Unclear	Unclear	Unclear	Low	Low	Low	Unclear
Tijink et al., 2009 (42)	Unclear	Low	Unclear	Unclear	Unclear	Unclear	Unclear	Low	Low	Low	Unclear
Song et al., 2017 (38)	Unclear	Low	Unclear	Low	Unclear	Unclear	Unclear	Low	Low	Low	Unclear
Lumen et al., 2022 (43)	Unclear	Low	Unclear	Low	Unclear	Unclear	Unclear	Low	Low	Low	Unclear

### 3.1 PD-L1-targeted immunoPET

In recurrent or metastatic HNSCC, durable responses to PD-1/PD-L1 inhibitors mainly occur in patients with high tumor PD-L1 expression. However, PD-L1 expression is variable, meaning single biopsies may not capture the complete tumor immune landscape, leading to underestimation of response potential. Through molecular imaging, radiolabeled tracers enable non-invasive, whole-body visualization of PD-L1 expression, providing a comprehensive, real-time assessment of tumor immune status that surpasses biopsy limitations (35).

Building on this rationale, a PD-L1-targeted immunoPET study by Verhoeff et al. (35) evaluated the Zirconium-89–Deferoxamine–durvalumab (^89^Zr-DFO-durvalumab), which consists of durvalumab, a human anti-PD-L1 mAb, that binds explicitly to PD-L1 expressed on tumor and immune cells. The tracer demonstrated high radiochemical purity, with a purity of at least 95%. A multicenter study utilized this tracer. Dose-finding identified 10 mg as the optimal dose, producing the highest mean tumor-to-blood (T:B) ratio and guiding subsequent imaging protocols. With immunoPET, 33 patients were scanned 5 days after injection. The study confirmed safety and tumor delineation but revealed high intra- and inter-patient variability in uptake, with no significant correlation to tumor PD-L1 expression or treatment response at the patient level. However, lesional uptake modestly correlated with FDG Standardized Uptake Value (SUV) peak, and elevated FDG total lesion glycolysis (TLG) was associated with poorer outcomes. Strengths include a multicenter design, harmonized protocols, sample cohort size, prospective dose optimization, and quantitative analyses using SUVpeak and T:B ratios, as well as FDG metrics. Limitations included reliance on archival PD-L1 biopsies, which may reduce the reliability of the correlation, and a single late imaging timepoint that restricts the assessment of uptake dynamics. These findings underscore the need for further refinement of PD-L1 PET tracers to better predict and stratify patients for HNSCC immunotherapy.

### 3.2 EGFR-targeted immunoPET

EGFR was selected as a target because it is highly expressed in 90-95% of HNSCC, is associated with radioresistance, and has a poor clinical prognosis (36). Beyond identifying tumor burden, EGFR also modulates immune responses, including the expression of PD-L1 and immune cell infiltration, and is considered a surrogate marker for immune profiling (37). Cetuximab, a chimeric anti-EGFR IgG1 approved by the FDA for HNSCC treatment, binds to EGFR, blocks its signaling pathways, and induces immune-mediated tumor cell death (38). However, the effectiveness of cetuximab depends on EGFR status: in tumors lacking EGFR expression, response to the drug is generally resistant regardless of accessibility, whereas in EGFR-overexpressing tumors, accessibility is expected to play a key role in determining drug uptake and treatment efficacy (39). Clinical trials have demonstrated that combining cetuximab or cisplatin with radiotherapy might achieve better patient outcomes than radiotherapy alone (38). The immunological characteristics of EGFR could justify its integration into immunoPET strategies, not only as a tumor biomarker but also as an immunologic imaging target. In a preclinical study by Song et al. (38), the radiolabeled Copper-64-*p*-SCN-Bn-PCTA-cetuximab (^64^Cu-PCTA-cetuximab) was employed to target EGFR in HNSCC models. The cellular uptake of ^64^Cu-cetuximab correlated with EGFR expression, as quantified by western blot and flow cytometry, across diverse HNSCC cell lines, validating target specificity. To closely mimic challenging clinical situations, the SNU-1066 xenograft model, characterized by high EGFR expression and resistance to cetuximab, was selected. Biodistribution studies initially revealed elevated uptake in the blood and liver at 2 hours post-injection, which subsequently decreased, whereas tumor uptake steadily increased, peaking at 48 hours post-injection. Specificity for EGFR was then confirmed by blocking experiments wherein an excess dose of cetuximab reduced tumor uptake by 48.8% in biodistribution analyses and by 56.7% in PET imaging, confirming tracer-target engagement. The immunoPET tracer exhibited a high radiolabeling yield and radiochemical purity of more than 98%. PET scans of 4 mice were acquired at 2, 24, 48, and 72 hours post-injection, each for 60 minutes. Imaging results were consistent with biodistribution data and digital whole-body autoradiography (DWBA), collectively demonstrating consistent tumor targeting by ^64^Cu-cetuximab. Compared to ^89^Zr, which has a longer half-life but delivers about eight times the whole-body radiation dose, ^64^Cu offers more favorable characteristics for immunoPET, including a lower radiation burden and a suitable half-life for antibody fragments, making it the preferred choice for the study. Despite these, translational relevance is moderated by certain limitations, including a limited sample size that restricts generalizability and a lack of specification regarding standardized uptake values (mean, maximum, or peak). Although these factors do not compromise the demonstrated robustness of radiosynthesis or target specificity, addressing them will be essential to fully establish the quantitative rigor and clinical applicability of the immunoPET approach.

In a multicenter study by Even et al. (39) of locally advanced LA-HNSCC, ^89^Zr-cetuximab PET/CT was employed, with imaging performed on 17 patients at two time points: 3 and 6 days or 4 and 7 days post-injection. Patients with stage III-IV LAHNSCC received a loading dose of unlabeled cetuximab to improve the blood bioavailability of the radiolabeled antibody and minimize hepatic uptake, followed by administration of ^89^Zr-cetuximab. The radiochemical purity of the tracer was approximately 98%, which enhances the robustness of the quantitative analyses. The study applied multiple uptake metrics, including SUVmean, SUVmax, SUVpeak, and tumor-to-background ratio (TBR), and harmonized imaging across different PET/CT scanners, significantly strengthening quantitative analysis. Tumor accessibility to cetuximab, a feature that was undetectable by FDG PET, was evident, with significant interpatient variability in uptake. Notably, TBR increased significantly in the second scan compared to the first, indicating improved image contrast and quantification at later time points. Although SUVmean and SUVpeak values differed statistically between tumors with low and high EGFR expression, TBR showed no significant difference by EGFR status. Moreover, the lack of correlation between FDG SUVpeak and ^89^Zr-cetuximab SUVpeak reinforces the distinct biological information provided by this immunoPET modality. ^89^Zr was chosen for labeling cetuximab for its long half-life (78 hours), matching cetuximab’s pharmacokinetics (69–95 hours), and as the author claimed that prior studies confirm the feasibility and safety of this approach in early human investigations, and recommended imaging at 6–7 days post-injection to optimize TBR uptake for future clinical applications. Limitations include the small patient cohort, variable scan timing, and potential effects of unlabeled cetuximab pre-dose on tracer biodistribution, which collectively moderate the robustness and clinical generalizability of the findings.

### 3.3 Tenascin-C-targeted immunoPET

The extradomain A1 of tenascin-C is a non-internalizing extracellular matrix protein highly expressed in multiple tumor types, including HNSCC, and represents an appealing imaging target. The Phase 0 study used iodine-124 (^124^I) as the positron-emitting isotope, which is well-suited for non-internalizing targets. To reduce free iodine uptake, patients received oral thyroid blockade with potassium perchlorate. Immunohistochemistry (IHC) confirmed the expression of tenascin-C in all tumors before imaging. In this Phase 0 microdosing immunoPET study, the radiochemical purity exceeded 97%, supporting the technical robustness of tracer preparation. Four patients received ^124^I-F16SIP, with PET/CT scans performed at 30 minutes and 24 hours post-injection. Initial uptake was observed in the liver, spleen, kidneys, and bone marrow, with a gradual decrease over time, while tumor uptake increased, making all primary lesions clearly visible by 24 hours. Tumor-involved lymph nodes were not detected. Ex vivo biopsies further demonstrated selective tracer accumulation in tumors, and blood/plasma sampling validated pharmacokinetics. The study confirmed feasibility but had limitations, including a small sample size, a lack of lymph node detection, interpatient variability, reliance on %ID/kg instead of SUVs, and an unclear clearance pathway, which may limit comparability and clinical translation (40).

### 3.4 ED-B-targeted immunoPET

ED-B is one isoform of fibronectin recognized as a critical angiogenic marker. It is highly expressed in both primary tumors and metastatic lesions, making it an appealing target for non-invasive tumor detection as well as for monitoring and predicting the response to anti-angiogenic therapies. The L19 antibody fragment, isolated from a phage display library, specifically binds ED-B with high affinity. Various L19-based immunoconjugates have been developed, including a dimeric single-chain variable fragment ((scFv)2), a human bivalent SIP of approximately 80 kDa, and a complete human IgG1 antibody. Among these, radioiodine-labeled L19-SIP and its derivatives are commonly used for imaging of ED-B expression (41).

Tijink et al. (42) evaluated the efficacy of ^124^I-labeled L19 for PET imaging of tumor angiogenesis and as a non-invasive scouting tool to guide ¹³¹I radioimmunotherapy (RIT). The study on nine HNSCC xenografts of varying sizes, allowing for the assessment of tracer detection sensitivity. Biodistribution studies were concordant for both ^124^I- and ¹³¹I-labels, with ^124^I-L19-SIP showing comparable TBR as found for ¹³¹I-L19-SIP. The ^124^I-L19-SIP conjugate demonstrated high radiochemical purity (99.9%), and thyroid uptake was blocked. Attenuation correction was performed using ^137^Cs transmission scans, a technique now largely replaced by more advanced techniques. PET imaging scans were performed at 24 and 48 hours post-injection, clearly visualizing tumors as small as 50 mm³, with early background activity, mainly due to partial deiodination, which had almost disappeared 48 h after tracer injection. The main limitations were the relatively small sample size, rapid renal clearance, and transient stomach/bladder activity due to catabolite excretion. However, further studies are needed to demonstrate that this tracer is suitable for imaging ED-B domain expression in patients using PET.

### 3.5 CD44v6-targeted immunoPET

An anti-CD44v6 chimeric mAb (cmAb), U36, was selected for the study because it has shown high and selective tumor uptake in HNSCC patients, and it internalizes into cells only to a limited extent. CD44v6 is expressed in only a few normal epithelial tissues (e.g., thyroid and prostate gland). Yet, it plays a significant role in the growth and development of solid tumors and metastasis. In HNSCC, over 96% of tumors exhibit CD44v6 expression in at least 50% of the cells. Beyond squamous cell carcinomas, CD44v6 is overexpressed in adenocarcinomas and ovarian cancer and in hematological tumors. Several research groups have visualized CD44v6 expression in tumors using U36 or its variants after radiolabeling them with different long-living radionuclides (43).

Verel et al. (44) developed a method to label antibodies with ^89^Zr using a TFP-activated N-succinyldesferrioxamine (N-sucDf) ester that forms a stable amide bond to lysine ϵ-amines. cmAb U36 was subsequently modified with N-sucDf and radiolabeled with ^89^Zr. *In vitro* studies demonstrated superior serum stability of cmAb U36-N-sucDf-^89^Zr compared to reference conjugates with succinimide ring-thioether linkers, which undergo radiolabel release and serum protein interactions leading to aggregation. The conjugate’s *in vivo* performance was assessed in 12 mice bearing HNX-OE xenografts derived from HNSCC cell lines. Radiochemical purity exceeded 95%, and PET imaging at 24, 48, and 72 hours post-injection clearly visualized all tumors as hot spots. Tumors as small as 19 mg were detectable with high TBR contrast, with nonspecific accumulation limited mainly to the blood pool in the heart and liver (and the nose at 24 hours). The novel conjugate exhibited slower blood clearance and higher tumor uptake compared to the reference conjugate, which showed faster blood clearance and increased colon content. Attenuation correction was performed using ^137^Cs transmission scans. These findings, while based on a limited animal cohort, show the practicality and reproducibility of this ^89^Zr-labeling approach, underscoring its potential for clinical immunoPET applications combined with RIT for sensitive tumor detection and patient stratification.

Building upon this, Verel et al. (45) applied their previously developed ^89^Zr-labeling method to evaluate ^89^Zr-labeled U36 immunoPET as a quantitative, *in vivo* surrogate for Yttrium-90 (^90^Y) RIT in a preclinical mouse model of HNSCC. Biodistribution analysis indicated that ^89^Zr-U36 closely matched the uptake pattern of ^88^Y-U36 across tumor tissue, blood, and most normal organs over time, with only minor late differences observed in sternum and thighbone, along with small but consistent differences in kidney and liver, attributed to radionuclide-chelator interactions. The radiotracer demonstrated a radiochemical purity of more than 97%. PET imaging of 12 mice at 24, 48, and 72 hours post-injection visualized millimeter-scale tumors, with image-derived tumor uptake values agreeing wellp with ex vivo gamma counting after partial-volume correction. Phantom test further demonstrated the high linearity of ^89^Zr PET activity quantification within relevant activity ranges, with expected nonlinearity at higher activities. Attenuation correction was used with ^137^Cs transmission scans. Spatial resolution and hot-sphere recovery coefficients were comparable to those of ^18^F, although a slightly lower recovery was noted for ^89^Zr due to its positron physical properties. The congruent physical half-lives of ^89^Zr (78.4 hours) and ^90^Y (64.1 hours), together with similar intracellular residualization profiles, provide a rationale for using ^89^Zr immunoPET as a practical, non-invasive tool for scouting and dosimetry in ^90^Y-RIT patient selection. Despite a relatively limited animal cohort and the need for further clinical validation, these findings support the utility of immunoPET as a surrogate for RIT dosimetry and patient stratification in antibody-based oncology therapies.

Expanding on these findings, Verel et al. (46) evaluated ^124^I-labelled cmAb U36 from radiolabeling through PET imaging as a pre-therapy scout for ¹³¹I-RIT in HNX-OE xenografts. Radiolabeling achieved more than 95% radiochemical purity, and thyroid uptake was blocked. Co-injection of ^124^I-cMAb U36 and ¹³¹I-cMAb U36 resulted in comparable tissue uptake values, with blood activity diminishing over time and tumor uptake increasing. Selective tumor uptake was confirmed with PET imaging of 12 mice at 24, 48, and 72 hours post-injection, which detected 15 out of 15 tumors, with minimal non-target signal limited to the cardiac blood pool, liver, and nose. Attenuation correction was used with ^137^Cs transmission scans. These findings support ^124^I immunoPET as a scouting tracer for ¹³¹I RIT. Overall, ^124^I-U36 demonstrated effective tumor targeting with promising clinical implications for immunoPET and treatment planning.

Following this, a preclinical proof-of-concept study targeting CD44v6 was conducted, evaluating two immunoPET strategies: direct targeting with [^89^Zr]Zr-3–TCO–U36 and a pretargeting approach with TCO–U36, followed 24 or 48 hours later by [^89^Zr]Zr-DFO-PEG_5_-Tz ([^89^Zr]Zr-3). The radiochemical purity was greater than 98%, demonstrating high technical quality. For each four mice, PET/CT imaging was performed at strategically selected time points tailored to each protocol: 1, 24, 48, and 71 hours post-injection for the direct targeting group; 25, 48, and 71 hours for the 24-hour pretargeting group; and 49 and 71 hours for the 48-hour pretargeting group. Although the direct group demonstrated higher tumor uptake, the pretargeting groups demonstrated comparable TBRs due to the rapid clearance of unbound tracer. Significantly, in the pretargeting approach, they reduced absorbed doses to the liver, spleen, and bone marrow, underscoring their dosimetric advantage. ^89^Zr was chosen for both approaches due to its long half-life, which enables extended imaging and direct comparison. The PET/CT results closely matched ex vivo biodistribution, demonstrating the specific accumulation of [^89^Zr]Zr-3–TCO–U36 in HNSCC xenografts and validating the specificity and feasibility of the pretargeted approach. However, the robustness of the study was hardened by a small cohort, characterized by variable tumor volumes, intratumoral heterogeneity, and broad SUVs. Overall, these results indicate pretargeted immunoPET as a promising approach to reduce patient radiation dose without compromising imaging contrast, while also enabling the prospective use of radionuclides with shorter physical half-lives in future clinical applications (43).

The first-in-human ^89^Zr-immunoPET study was conducted in 2006 by Börjesson et al. (47), who evaluated the ^89^Zr-labeled cmAb U36 in 20 HNSCC patients. The radiochemical purity was always more than 94.9%. Whole-body scans were acquired at approximately 1, 24, 72, and 144 hours post-injection. Notably, CD44v6 expression was not biopsy-confirmed in this cohort, as prior evidence had established, rendering additional confirmation unnecessary, as they mentioned. Early scans showed mainly blood pool activity with delineation of nose, heart, lungs, liver, spleen, and kidneys, which diminished over time. Tumor uptake increased over time, demonstrating high accuracy for detecting both primary tumors and lymph node metastases. Transmission scans were performed using germanium-68 (Ge^68^) rods. ImmunoPET performance was comparable to Computed Tomography (CT) and Magnetic Resonance Imaging (MRI); some lymph nodes that CT/MRI missed were also small and contained little tumor tissue. In a subset of six patients, the diagnostic accuracy was comparable to that of ^18^F-FDG PET, further validating the potential of CD44v6-targeted imaging. The study was limited by the absence of SUV-based quantification and reliance solely on visual assessment. Additionally, the modest sample size and the occurrence of human anti-chimeric antibody responses in some patients, despite overall safety and tolerability, are of concern. Taken together, these findings provide preliminary support for the feasibility of CD44v6-targeted ^89^Zr-immunoPET in HNSCC.

Since no radiation dose estimates had been reported in earlier studies with ^89^Zr-cmAb U36 or other ^89^Zr-labeled mAbs, the same group (48) later conducted a dosimetry and quantification study in the previously imaged patient cohort. They found that PET-based quantification of left ventricular activity correlated well with blood samples, except in overweight patients, particularly in later images. Similarly, PET-derived tumor uptake aligned closely with biopsy measurements. Tumor uptake increased over time, sometimes accompanied by thyroid uptake. Image quality varied among patients. Organ dose estimates showed the highest absorbed dose in the liver, followed by the kidneys, thyroid, lungs, and spleen. Renal excretion was minimal (<3% in the first 72 h). These findings suggest the use of ^89^Zr-cmAb U36 immunoPET for assessing antibody biodistribution, which enables patient stratification based on tumor uptake and aids in treatment planning. However, limitations included the exclusion of several patients from quantitative analysis, and the mean effective dose was approximately 40 mSv, which restricted the number of repeated scans. The authors stated that lower injected doses and newer PET/CT technology could reduce radiation exposure in future studies.

**Figure 4:**
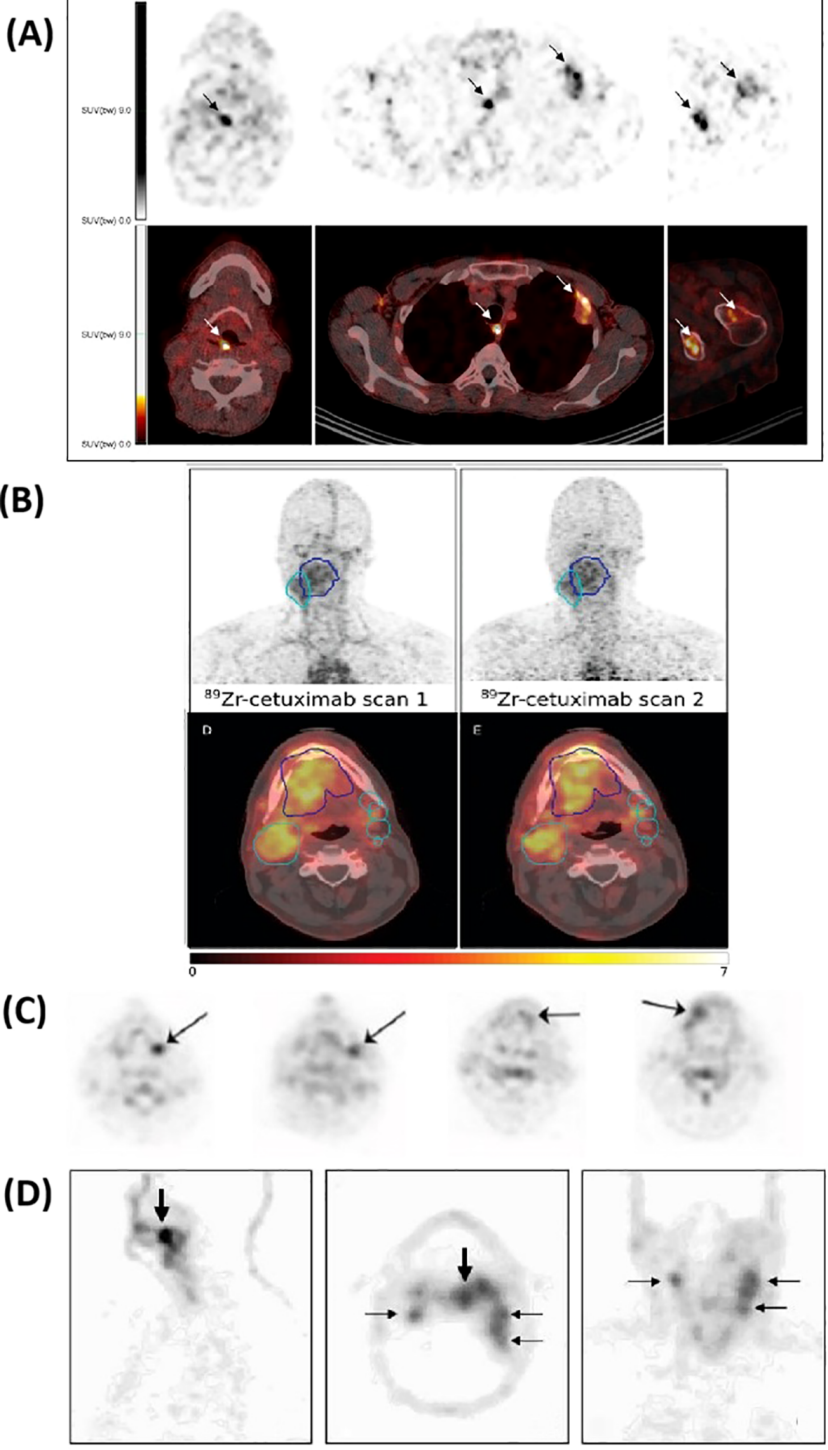
Representative clinical immunoPET images using different tracers. The top panel **(A)** shows ^89^Zr-DFO-durvalumab PET/CT in two patients, where arrows indicate focal tracer accumulation. Additional images display uptake in lymph nodes, pleura, and bone lesions for 5 days post-injection. Adapted from Verhoeff et al. (35). The second panel **(B)** presents ^89^Zr-cetuximab PET/CT images at 4 and 7 days post-injection; the gross tumor volume (GTV) is delineated in blue, and the clinical target volume (CTV) is shown in cyan. Adapted from Even et al. (39). The third panel **(C)** illustrates the uptake of ^124^I-F16SIP 24 hours post-injection in four patients. Adapted from Heuveling et al. (40). The bottom panel **(D)** shows ^89^Zr-cmAb U36 of an HNSCC patient with a tumor in the left tonsil (large arrow) and lymph node metastases (small arrows) at the left (level II and III) and right (level II) side of the neck. Images were obtained 72 hours post-injection. Adapted from Borjesson et al. (47).

**Figures 4** presents representative immunoPET images from the reviewed clinical studies of HNSCC, illustrating tracer accumulation patterns and target specificity.

## 4 Discussion

To the best of our knowledge, this is the first review to explore the current landscape of immunoPET imaging in HNSCC, highlighting immune-specific targeting radiotracers and emerging preclinical and clinical applications. The reviewed studies investigated five distinct immune targets: PD-L1, EGFR, Tenascin-C, and ED-B, as well as CD44v6, and utilized three radiotracers: ^89^Zr, ^124^I, and ^64^Cu.

ImmunoPET imaging provides high specificity and sensitivity for detecting primary tumors, lymph node involvement, and distant metastases, serving as a potential complement to IHC when biopsies are not feasible. It enables precise patient stratification; positive findings can guide antibody or antibody-based RIT, while negative or heterogeneous cases may necessitate a multidisciplinary treatment approach. ImmunoPET also enhances early disease triage and facilitates image-guided surgical interventions. Notably, it advances immunotherapy by dynamically visualizing immune responses and predicting outcomes to immune checkpoint inhibitors (28). Beyond immunotherapy, immunoPET demonstrates clinical relevance in combination with radiotherapy by assessing synergistic effects such as the abscopal effect, driven by immune activation. Furthermore, the integration of immunoPET with metabolic imaging enhances tumor characterization and treatment evaluation, a role further strengthened by texture analysis and artificial intelligence-based image interpretation (49).

In HNSCC, the only published study targeting PD-L1 with immunoPET is the ^89^Zr-DFO-durvalumab study, which demonstrated feasibility and safety, with detectable tumor and immune cell PD-L1 uptake across patients. However, uptake demonstrated substantial intra- and inter-patient heterogeneity and did not correlate significantly with PD-L1 expression or treatment response at the patient level. Only modest correlations were observed with FDG SUVpeak and the association of elevated FDG TLG with poorer outcomes, underlining the complex interplay between metabolic and immune features in HNSCC. Limitations, including a small cohort size, the potential influence of unlabeled durvalumab co-dosing, reliance on archival biopsy data, and a single late imaging time point, highlight areas that require further investigation. Nonetheless, these results suggest that ^89^Zr-DFO-durvalumab immunoPET holds promise for non-invasive, whole-body assessment of PD-L1 status. However, additional refinement and validation are essential to establish its predictive value and clinical utility (35). By contrast, a ^89^Zr-atezolizumab study in patients with bladder cancer, non-small cell lung cancer (NSCLC), and triple-negative breast cancer (TNBC) reported that pretreatment immunoPET uptake correlated more strongly with clinical outcomes than PD-L1 IHC or RNA-sequencing based gene signatures, with higher tracer uptake predicting improved progression-free and overall survival (50). Unlike the HNSCC experience, in which predictive value was limited, this trial suggested that PD-L1 immunoPET can outperform conventional biopsy-based biomarkers in certain tumor settings. Clinically, this uncertainty is critical, as approximately 29% of R/M HNSCC patients experience hyperprogression and rapid tumor progression (≥2-fold growth kinetics) following PD-1/PD-L1 blockade, often associated with regional recurrence and reduced progression-free survival (51). Therefore, reliable predictive tools are needed to avoid harm to susceptible individuals.

EGFR is overexpressed or otherwise activated in the majority of HNSCC and is linked to radioresistance (36), making it a compelling target for imaging and therapy. As a member of the receptor tyrosine kinase (RTK) family, it represents one of the most studied oncogenic drivers. Targeted therapies, such as tyrosine kinase inhibitors (TKIs) and mAbs, have been developed to inhibit RTK signaling, and immunoPET imaging now enables non-invasive assessment of EGFR expression and heterogeneity (28). This may explain why two of the 11 reviewed studies selected EGFR for immunoPET evaluation. Song et al. (38) demonstrated the specificity and translational potential of ^64^Cu-cetuximab as an EGFR-targeted immunoPET tracer. Their validation, which combined western blot, flow cytometry, biodistribution blocking studies, and DWBA, confirmed tracer engagement with EGFR-expressing tumors. Notably, the choice of ^64^Cu was strategically justified due to its optimal half-life, which is compatible with antibody kinetics, and a substantially lower radiation dose compared to ^89^Zr, thereby reinforcing its suitability for clinical immunoPET imaging. These findings may validate ^64^Cu-cetuximab as a promising tool for precise tumor detection, also paving the way for its integration into personalized oncologic assessment and therapy monitoring. However, limitations such as a small sample size and a lack of standardized SUV reporting must be addressed to strengthen its clinical applicability.

Building on the promising preclinical results with ^64^Cu-cetuximab, the multicenter clinical study by Even et al. (39) demonstrated the feasibility and complexity of ^89^Zr-cetuximab PET/CT in locally advanced HNSCC, making essential progress in clinical immunoPET imaging. The use of unlabeled cetuximab as a loading dose to enhance tracer bioavailability and reduce liver uptake underscores a critical strategy for optimizing *in vivo* targeting and improving image quality. Harmonized protocols across centers, along with the use of multiple standardized uptake metrics, enhanced quantitative reliability. The observed increase in TBR later supported the use of delayed imaging for optimal contrast and accurate tumor delineation. However, significant interpatient variability and unexpectedly high uptake in some cases, despite low EGFR levels, reveal challenges in interpreting uptake. The unclear best metric for quantifying uptake SUVmax, SUVpeak, SUVmean, or TBR highlights the need for standardization and interpretation. Interestingly, the lack of correlation between FDG PET and ^89^Zr-cetuximab uptake highlighted the complementary biological information of immunoPET beyond metabolic activity. Despite limitations such as small sample size, variable scan timing, and potential pre-dosing effects, the study represents a meaningful advance toward refining EGFR-targeted immunoPET for personalized cancer assessment.

All immunoPET tracers reviewed were full-length mAbs, except for ^124^I-L19-SIP and ^124^I-F16SIP, which are smaller antibody fragments (SIP format) engineered for faster clearance and improved tumor penetration. Tenascin-C imaging represents a distinct approach, utilizing a small antibody fragment, to achieve faster kinetics and reduced background. Tenascin-C-targeting imaging using ^124^I-F16SIP demonstrated selective tumor localization and good tolerability. All tumors were visualized 24 hours post-injection, though only one was faintly visible at 30 minutes. Notably, PET imaging missed tumor-involved lymph nodes, highlighting limitations in spatial resolution and tracer sensitivity for identifying micrometastases. Additionally, the small cohort size, interpatient variability, and the absence of standardized SUV quantification in the analysis limit the robustness of the findings. Despite these limitations, this finding supports further investigation of tenascin-C as a target and ^124^I-F16SIP as a diagnostic agent (40). While ^124^I-L19-SIP enables the sensitive detection of angiogenic HNSCC xenografts, with biodistribution closely matching that of therapeutic ¹³¹I-L19-SIP, and tumors as small as 50 mm³ are clearly visualized. These findings support its potential as a non-invasive scouting tracer for RIT. However, rapid renal clearance, partial deiodination with early background activity, and a limited sample size constrain the translational relevance and generalizability of the results. However, technically promising, clinical validation is necessary to confirm the robustness of ED-B targeting, optimize imaging windows, and benchmark it against alternative labeling strategies (42).

On the other hand, CD44v6 has emerged as a prominent and well-studied target in immunoPET imaging of HNSCC, consistently identified in more than half of the reviewed studies. Preclinical work, led primarily by Verel et al. (44), demonstrated that radiolabeling the anti-CD44v6 antibody U36 with ^89^Zr using optimized N-sucDf chemistry yielded stable conjugates with favorable pharmacokinetics, enabling sensitive detection of small tumors with high TBR. These preclinical validations extended to quantitative imaging as surrogates for RIT, where ^89^Zr-U36 closely mirrored the biodistribution and dosimetry of ^88^Y-U36, underscoring its theranostic potential (45). Parallel evaluation of ^124^I-labeled U36 further highlighted its utility as a pre-therapy PET scout for ¹³¹I RIT, reinforcing the role of CD44v6 in theranostic applications (46). Efforts to improve safety through pretargeting strategies have reduced the off-target radiation dose while maintaining TBR comparable to those of direct targeting, suggesting promise for future directions, particularly with shorter-lived radionuclides (43). Clinically, the first-in-human ^89^Zr-U36 immunoPET study demonstrated high accuracy in delineating primary tumors and metastatic lymph nodes, outperforming CT and MRI (47). Notably, the mean effective radiation dose observed in the early clinical study was approximately 40 mSv (48), which is higher than the standard diagnostic PET examinations, with an average effective dose reported at 6.7 mSv (52). This necessitates careful consideration of scan frequency and motivates future improvements through lower injected activities and advanced PET/CT technology. Collectively, CD44v6-targeted immunoPET exemplifies a robust biomarker platform combining tumor specificity, quantitative imaging capacity, and translational readiness for personalized antibody-based diagnostics and RIT. While challenges remain, including small clinical cohorts and relatively high radiation doses in early studies, the accumulated evidence supports CD44v6 as a key biomarker for advancing precision oncology imaging and patient stratification strategies in HNSCC.

Collectively, these 11 studies demonstrate the promise and the heterogeneity of immunoPET in HNSCC, with tracer uptake varying widely across targets and protocols. The findings underscore the need for standardized imaging methods, standardized quantitative metrics, and adequately powered cohorts to ensure reproducibility and clinical relevance. While still in its early stages of integration, immunoPET holds potential for patient stratification, early treatment monitoring, and reducing reliance on invasive biopsies. Study sample sizes were small (4–33), limiting generalizability and statistical power. Consistent with prior immunoPET work in lymphoma, the available studies were limited by small patient cohorts and protocol features, such as unlabeled antibody preloading, which demonstrably alters biodistribution and tumor targeting. In addition, imaging-tissue discordance, driven by intratumoral heterogeneity, has complicated the validation of immunoPET against histology (53).

**Figure 5:**
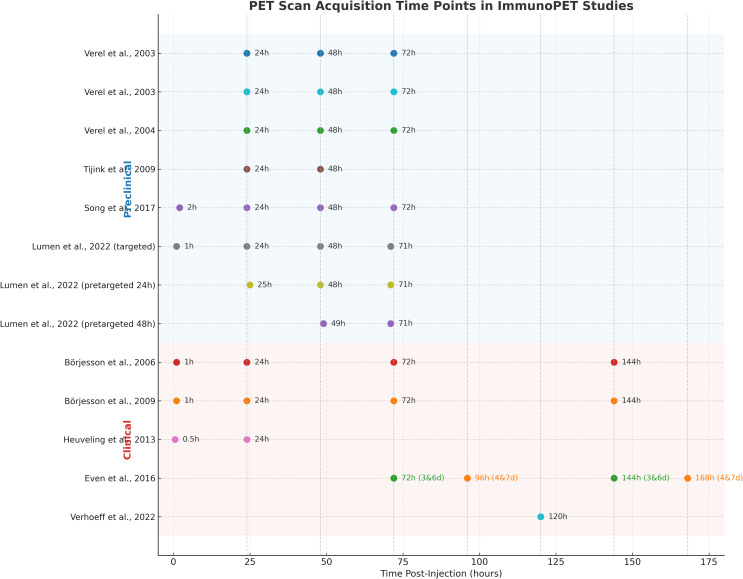
PET scan acquisition time points for each tracer used in the reviewed immunoPET studies. Imaging intervals are shown along the x-axis post-injection for each radiotracer and research.

Notably, all reviewed preclinical studies employed subcutaneous implants, which are convenient and reproducible but can differ significantly from orthotopic HNSCC in terms of vascular function, stromal architecture, and immune suppression (54). These differences may overestimate tracer uptake and underrepresent site-specific barriers, limiting translational inference. The geographic distribution of studies is highly concentrated, with all conducted in the Netherlands and one in South Korea. The absence of studies from other regions, such as North America, Africa, and other parts of Asia and Europe, raises concerns about the global applicability of the findings and underscores the need for more comprehensive international exploration. Another gap across studies is that none provided a clear cut-off threshold for PET-derived parameters to stratify responders and non-responders. The lack of consensus on which PET measures best reflect drug delivery or target accessibility introduces further complexity to interpretation. Moreover, dynamic PET imaging or detailed pharmacokinetic modeling was not explored, limiting insight into the temporal dynamics of the immune response. Variations in scan timing, tracer dosing, and image quantification techniques further limited comparability across studies. These methodological inconsistencies hinder the establishment of best practices in immunoPET. **Figure 5** summarizes the scan acquisition time points used across the 11 reviewed studies, ranging from early imaging at 30 minutes to delayed imaging up to 7 days post-injection. These variations may reflect differences in tracer pharmacokinetics, with early scans often showing higher background activity, whereas later scans provided improved tumor-to-background contrast.

While the reviewed studies have primarily focused on tumor-associated antigens such as PD-L1, EGFR, Tenascin-C, ED-B, and CD44v6, recent advances in immunoPET have expanded the field toward imaging functional immune dynamics, particularly T-cell activation and exhaustion. T-cell lineage markers, such as CD3, CD4, and CD8, provide valuable insights into the mobilization and infiltration of T cells; however, they do not reveal the functional state of these cells (55). Distinguishing between activated and exhausted T cells is critical for accurately assessing immune response within the TME (56). ImmunoPET imaging of activation-specific biomarkers offers a non-invasive approach to evaluating T-cell function. Markers such as inducible T-cell costimulator (ICOS), CD134 (OX40), and cytokines like IFN-γ and IL-2 are selectively upregulated upon T-cell activation and have been successfully targeted with radiolabeled antibodies in preclinical models. For instance, ^89^Zr-labeled anti-ICOS mAbs have been used to monitor the activation, expansion, and tumor retention of CD19-directed CAR T-cells in murine models of B-cell lymphoma (55).

In contrast, chronic antigen stimulation in the context of cancer or persistent infections drives CD8^+^ effector T cells (Teff) into an exhausted state (Tex), characterized by diminished effector function and proliferative capacity. This exhaustion, exacerbated by the immunosuppressive TME, is represented by the expression of inhibitory receptors, such as PD-1, LAG-3, TIM-3, CTLA-4, BTLA, VISTA, and TIGIT. Recent advancements in immunoPET have enabled the visualization of these exhaustion-associated pathways, facilitating the characterization of dysfunctional immune responses (55). Therefore, delineating T-cell activation and exhaustion states may provide superior prognostic value than conventional quantification of immune cell infiltration alone, particularly in distinguishing functional immunity from immune anergy (56).

Only two of the 11 included studies reported SUVpeak, despite its superior robustness over SUVmax, particularly in small lesions (57–60). This could limit quantitative standardization and comparability of response evaluation across immunoPET studies. Moreover, all radiotracers used in the reviewed studies: ^64^Cu, ^89^Zr, and ^124^I, have long-lived isotopes (**Table 4**), which are well-suited for antibody-based imaging (61). However, their prolonged half-lives can make clinical scheduling more challenging, particularly for patients undergoing radiotherapy. None of the studies explored shorter-lived radionuclides such as ^18^F, which could offer lower radiation burden, better logistics, and compatibility with same-day imaging.

**Table 4:** Physical properties of the radionuclides used in the reviewed studies and ^18^F as a potential alternative [Adapted from Amgheib et al., 2022 (74)].

Radionuclide	Half-life	Decay mode (β+ mode %)	Position energy (MeV)
^18^F	1.82 h	97	0.65
^64^Cu	12.7 h	18	0.65
^89^Zr	78.4 h	23	0.91
^124^I	100.2 h	23	1.54

In addition to the potential efforts of shorter-lived PET radionuclides, it is also essential to consider the complementary role of SPECT-based immunoimaging, given its widespread availability and evolving capabilities. Antibody (Ab) radiolabeling was initially pioneered using SPECT radioisotopes such as ¹³¹I, ¹²³I, ¹¹¹In, and ^99m^Tc. Over time, the nuclear medicine community shifted its focus toward PET radioisotopes, such as ^89^Zr, ^64^Cu, and ^124^I, due to their improved availability, higher purity, and optimized nuclear production methods. PET imaging offers higher spatial resolution and sensitivity than SPECT, enabling more accurate quantification of tracer uptake. However, these advantages come with higher production costs and a greater radiation burden because of the higher photon energies of PET radionuclides. In clinical practice, this increased radiation exposure is often offset by the higher administered activity required for SPECT imaging, compensating for its lower detector sensitivity. Recent advances in detector and system design have improved sensitivity for both modalities, potentially allowing for lower administered doses and enhancing the feasibility of immunoPET and immunoSPECT imaging in the future. A unique feature of SPECT is its ability to perform dual-isotope imaging, as each SPECT radionuclide emits γ-rays with distinct energy signatures (62). This capability enables the simultaneous imaging of multiple targets, particularly when mAbs are labeled with radionuclides emitting different gamma energies. Dual-tracer immunoSPECT imaging has been demonstrated in clinical and preclinical settings. Technological advancements, such as cadmium zinc telluride (CZT) detectors, which provide superior energy resolution to conventional Sodium Iodide (NaI) detectors, and novel image reconstruction algorithms, have further enhanced this capability. While immunoPET generally offers greater imaging sensitivity, immunoSPECT remains particularly valuable in theranostic applications using agents like ^177^Lu or ^67^Cu, whose gamma emissions are compatible with SPECT scanners. Furthermore, immunoPET has enabled the visualization of immune processes, including T-cell exhaustion and the expression of immunosuppressive biomarkers within the TME. Meanwhile, SPECT continues to evolve, and the ability to simultaneously image two biological events non-invasively through dual-isotope techniques marks a promising direction for its application (55).

As demonstrated in one of the reviewed studies, the dual use of ^64^Cu-labeled antibodies for PET imaging and ^177^Lu-labeled antibodies for SPECT-based therapy in cetuximab-resistant HNSCC models highlights the complementary diagnostic and therapeutic roles of these modalities. In that study, ^64^Cu-PCTA-cetuximab enabled quantitative immunoPET imaging and patient selection, while ^177^Lu-PCTA-cetuximab delivered targeted radiation therapy, significantly reducing tumor volume and increasing apoptosis in resistant xenografts. This theranostic pairing also demonstrated favorable dosimetry and clear visualization on both PET and SPECT/CT scans, indicating that combining imaging and therapy enhances treatment planning and monitoring in cases of cetuximab resistance.

In accordance with PRISMA 2020 guidelines, several limitations in the review process should be acknowledged. First, only English-language, peer-reviewed publications were included, which may have excluded relevant studies in other languages or unpublished data, introducing potential publication bias. Additionally, the data extraction and study selection process, although performed systematically, is subject to potential reviewer bias and human error. These factors may influence the completeness and objectivity of the evidence synthesis presented in this review.

Future research should explore additional immune targets beyond PD-L1, EGFR, Tenascin-C, ED-B, and CD44v6 to represent the complex and heterogeneous TME better. Larger trials with standardized imaging protocols are needed to enhance reproducibility and enable meaningful cross-study comparisons. Validating findings in orthotopic or spontaneous models to better reflect the HNSCC microenvironment. Furthermore, developing next-generation radiotracers, especially those labeled with shorter-lived radionuclides such as ^18^F, should be prioritized to reduce radiation burden and facilitate same-day imaging workflows.

While full-size mAbs are highly specific, their large size limits tumor penetration and slows clearance. To overcome these limitations, smaller antibody fragments are being developed as radiotracers, offering improved tissue penetration, faster pharmacokinetics, and compatibility with short-lived PET isotopes, such as ^18^F, for efficient, same-day imaging (63). Although long-lived radionuclides such as ^89^Zr remain valuable in immunoPET due to their half-life compatibility with mAbs, they are limited by lower spatial resolution and image quality compared to ^18^F, mainly because of their longer positron range and lower branching ratios (64).

Among the emerging alternatives, ^18^F-FAraG presented significant potential as a non-invasive imaging tool for evaluating activated CD8+ T cells. At the molecular level, AraG enters cells via nucleoside transporters and is phosphorylated to its monophosphate form (AraGMP) by cytosolic deoxycytidine kinase (dCK) or mitochondrial deoxyguanosine kinase (dGK) (65). The Phosphorylated ^18^F-FAraG accumulates in activated T cells, allowing PET imaging (66). ^18^F-FAraG enables early monitoring of immune activation and predicts therapeutic outcomes. Clinical trials have demonstrated that changes in ^18^F-FAraG signal post-therapy correlate with patient survival, underscoring its potential for assessing early immune responses. As its clinical utility expands, ^18^F-FAraG could play a key role in personalized treatment strategies and monitoring immune-modulating therapies in oncology (67).

This underscores the need for improved detector technology and reconstruction algorithms, as well as the development of novel radiotracers and labeling approaches that combine favorable antibody pharmacokinetics with the superior spatial resolution of shorter-lived isotopes, thereby achieving high specificity and image quality. Future strategies should also emphasize the use of multi-target immunoPET panels to simultaneously assess multiple immune and tumor markers, thereby providing a more comprehensive view of tumor heterogeneity and evolution within the TME. In addition, integrating the immunome with advanced SPECT technologies may offer complementary advantages, particularly in theranostic applications and dual-isotope imaging. When evaluating potential candidates, it is essential to carefully consider their affinity, safety, specificity, sensitivity, immunogenicity, tissue penetration, clearance, and the associated radiation exposure to patients and staff.

A promising era for future research is the integration of radiomics and machine learning into immunoPET imaging workflows. Radiomics has demonstrated the ability to surpass conventional visual assessment, laboratory tests, and genomic or proteomic assays by linking imaging features with molecular, phenotypic, and genetic data to support clinical decision-making (68). In HNSCC, several studies have demonstrated the value of machine learning and radiomics in tumor stratification and prediction of response (69–72). Incorporating these tools into immunoPET has the potential to improve response prediction, guide treatment decisions, and facilitate individualized treatment planning. Despite these potential advantages, translating radiomics and AI into clinical practice remains challenging due to issues with reproducibility, standardization, interpretability, and validation (73).

Future work should also address gaps such as the lack of head-to-head tracer comparisons, the need for standardized immune-response imaging criteria, and the importance of ensuring reproducibility through open data sharing and harmonized protocols. Addressing logistical challenges, including tracer production, cost-effectiveness, and immunogenicity profiling, will be essential for broader clinical integration. As immunoPET advances toward clinical integration, overcoming methodological, technical, and logistical barriers will be necessary to fully harness its potential to support clinical decision-making and personalized treatment.

## 5 Conclusion

The findings of this review indicate that although immunoPET imaging holds promise in the management of HNSCC, its clinical application remains limited by significant challenges. Evidence remains restricted by small sample sizes, heterogeneous study protocols, lack of standardized imaging criteria, and variability in radiotracer selection. The reliance on long-lived isotopes contributes to a high radiation burden and limited global representation, further hindering clinical translation. As such, immunoPET is not yet ready for routine integration into workflows for risk stratification or treatment monitoring. Further research is needed to prioritize expanding immune target diversity, standardizing imaging protocols, and integrating shorter-lived tracers and radiomics workflows. Additionally, future strategies should explore multi-target imaging approaches and the complementary role of advanced SPECT technologies to facilitate broader clinical adoption.

## Data Availability

The original contributions presented in the study are included in the article/supplementary material. Further inquiries can be directed to the corresponding author.
